# Neurogenesis Is Increased in Human Neural Stem Cells by Aβ40 Peptide

**DOI:** 10.3390/ijms23105820

**Published:** 2022-05-22

**Authors:** Adela Bernabeu-Zornoza, Raquel Coronel, Charlotte Palmer, Alberto Martín, Victoria López-Alonso, Isabel Liste

**Affiliations:** 1 Unidad de Regeneración Neural, Unidad Funcional de Investigación de Enfermedades Crónicas (UFIEC), Instituto de Salud Carlos III (ISCIII), 28222 Majadahonda, Spain; raquelicoronel@gmail.com (R.C.); cpalmer248@gmail.com (C.P.); 2 Instituto de Investigación de Enfermedades Raras (IIER), Instituto de Salud Carlos III (ISCIII), 28222 Majadahonda, Spain; martin.alberto77@gmail.com; 3 Unidad de Biología Computacional, Unidad Funcional de Investigación de Enfermedades Crónicas (UFIEC), Instituto de Salud Carlos III (ISCIII), 28222 Majadahonda, Spain; victorialopez@isciii.es

**Keywords:** Aβ40, human neural stem cells, Alzheimer’s, neurogenesis, cell proliferation

## Abstract

Amyloid-β 40 peptides [Aβ1-40 (Aβ40)] are present within amyloid plaques in the brains of patients with Alzheimer’s disease (AD). Even though Aβ peptides are considered neurotoxic, they can mediate many biological processes, both in adult brains and throughout brain development. However, the physiological function of these Aβ peptides remains poorly understood, and the existing data are sometimes controversial. Here, we analyze and compare the effects of monomeric Aβ40 on the biology of differentiating human neural stem cells (human NSCs). For that purpose, we used a model of human NSCs called hNS1. Our data demonstrated that Aβ40 at high concentrations provokes apoptotic cellular death and the damage of DNA in human NSCs while also increasing the proliferation and favors neurogenesis by raising the percentage of proliferating neuronal precursors. These effects can be mediated, at least in part, by β-catenin. These results provide evidence of how Aβ modulate/regulate human NSC proliferation and differentiation, suggesting Aβ40 may be a pro-neurogenic factor. Our data could contribute to a better understanding of the molecular mechanisms involved in AD pathology and to the development of human NSC-based therapies for AD treatment, since these results could then be used in diagnosing the disease at early stages and be applied to the development of new treatment options.

## 1. Introduction

Aggregations of amyloid-β peptides (Aβ) in amyloid plaques, together with the development of neurofibrillary tangles, constitute the best-known histopathological marks in Alzheimer’s disease (AD), according to post-mortem studies of patient brains. AD is also characterized by other physical characteristics, such as the progressive impairment of cognitive functions, motor disturbances and progressive memory loss [1,2].

Aβ is a peptide with a length of 39–43 amino acids and with a molecular weight of 4 kDa. Aβ peptides are generated by proteolytic processing from amyloid precursor proteins (APP) in the form of monomeric peptide [3,4], but during aging, and in disorders such as AD, it accumulates and aggregates in fibers that precipitate in the form of plaques in the brain, causing toxicity [5,6].

Aβ peptides have been associated with toxic effects on neurons [7]. However, these peptides, which are also present in healthy adult brains, could mediate important physiological processes, such as the regulation of synaptic activity and neuronal survival, behaving as a neuroprotective element [8,9,10,11].

Furthermore, Aβ peptide is generated during embryonic cerebral development, and some studies have shown that Aβ is essential for correct brain development [12,13], suggesting that Aβ peptide is not always causing neurotoxicity, and it appears to have a neuroprotective effect [14].

Although the two main isoforms of Aβ (Aβ1–40 (Aβ40) and Aβ1–42 (Aβ42)) are present in amyloid plaques, Aβ42 peptide is known to be more abundant than Aβ40 within these plaques due to its greater capacity for self-aggregation [15]. In addition, a few studies have shown that Aβ42 is more toxic than Aβ40 both in vivo and in vitro [15]. On the other hand, the Aβ40 peptide is the major Aβ species generated by neurons in physiological conditions. In different pathologies like AD, the Aβ40 levels are diminished, which provokes a rise in the Aβ42/Aβ40 ratio [5,15] and the following increment of Aβ42 levels. In the last decade, several studies have begun to consider the effects of an aggregated Aβ40 peptide (oligomers or fibrils), since it is what is considered the pathological form of the Aβ peptide. Despite this, the Aβ40 peptide in its monomeric/soluble form is poorly understood. For that reason, studies on this line would be of great interest, since understanding its physiological function would help to understand what is happening to originate the aggregation of the Aβ peptides. Altogether, it would be helpful to advance in the search for therapies for the treatment of AD.

Despite the effort being made in the field, the AD has still not been cured. An alternative strategy being studied involves using endogenous or implanted neural stem cells (NSCs) to compensate for the neuronal degeneration in AD.

NSCs have the capacity to self-renew and to give rise to the main neural cell types of the central neural system (CNS) (neurons, astrocytes and oligodendrocytes). These cells can be obtained from fetal, neonatal or adult brains or from the differentiation of pluripotent stem cells (PSCs) [16,17]. Human NSCs provide a useful tool to clinically advance in stem cell-based therapies for several neurodegenerative disorders and have facilitated a better understanding of early human brain development and the molecular pathologies associated with neurodegeneration [18].

Previous studies have observed that monomeric Aβ peptide can have effects on the phenotypic specification and proliferation of both rat [19] and mouse neural precursor cells [20,21,22]. Similarly, a recent work from our group showed that monomeric Aβ42 increases the proliferation and astrogliogenesis in human NSCs [23]. However, it is not yet known whether the monomeric form of Aβ40 has any effect on the biology of human NSCs.

In this study, we used human NSCs of the hNS1 cell line. These cells are multipotent, clonal, derived from the developing human fetal telencephalon and v-myc-immortalized [24]. hNS1 cells have been extensively characterized in previous studies [24,25,26,27], and their properties are maintained over time; therefore, they may be a good tool in analyzing the effects of Aβ peptides.

The aim of the present work is to analyze and compare the effects of Aβ40 on the biology of human NSCs (i.e., cell survival/death, cell proliferation and phenotypic specification). To this end, we tested different doses of monomeric (soluble) Aβ40 peptide in differentiating hNS1 cells. These results, together with those previously obtained by our group, provide evidence that Aβ monomers affect the properties and biology of human NSCs and, therefore, could contribute to the advancement of therapies based on the use of human NSCs for the treatment of AD.

## 2. Results

**Figure 1:**
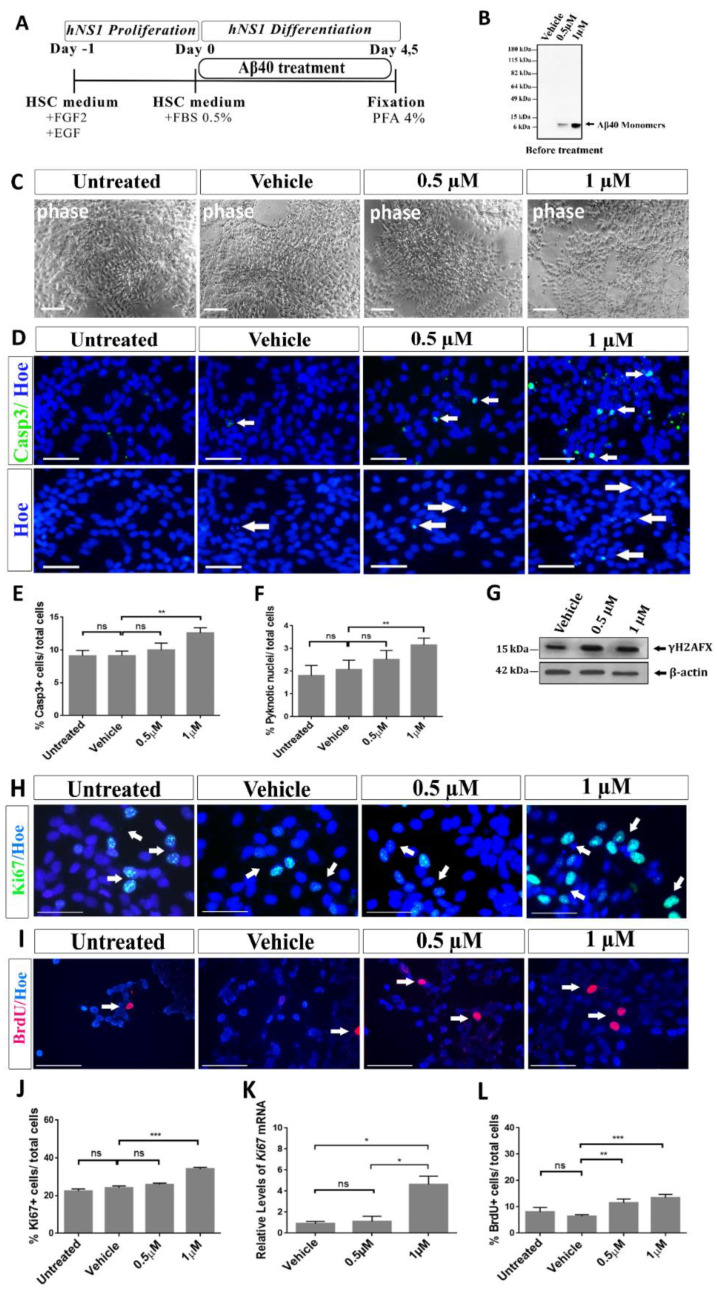
Aβ40 enhances cell death and promotes proliferation in differentiating hNS1 cells. (**A**) Schematic view of the hNS1 differentiation protocol (see the Materials and methods section). (**B**) Representative Western blot (WB; using 4G8 antibody) analysis of the Aβ40 forms at different concentrations (0.5 and 1 µM) present in the extracellular medium before the treatment with the Aβ40 peptide. (**C**) Representative phase contrast images of hNS1 cells treated with the different doses (0.5 µM and 1 µM) of the Aβ40 peptide and control groups (untreated and vehicle (DMSO) treated cells) for 4.5 days. (**D**) Representative images of activated caspase-3 immunoreactivity (upper panels; ActCasp3; green, see arrows). Representative images of fragmented nuclei stained with Hoechst (lower panels; Hoe; see arrows). Scale bars, 100 µm (**C**) and 50 µm (**D**). (**E**) Analysis of the percentage of ActCasp3+ cells/total cells after Aβ40 treatment. (**F**) Quantification of the percentage of fragmented nuclei in the different cell groups. (**G**) WB analysis of the γH2AFX (15 kDa) expression in cell extracts after treatment with the Aβ40 peptide. β-actin was used as a loading control (42 kDa). (**H**) Representative images showing Ki67 immunoreactivity (green, arrows). (**I**) Images for BrdU immunoreactivity (red, arrows). (**J**) Percentage of Ki67+ cells/total cells in the different cell groups. (**K**) Relative expression levels determined by the RT-qPCR analysis of *Ki67* mRNA. (**L**) Percentage of BrdU+ cells/total cells in the different experimental groups. Nuclei were stained blue with Hoechst. Scale bar, 50 µm. Data are represented as the mean ± SD of at least three different experiments (*n* = 3). Statistical significance of one-way ANOVA with Tukey’s post hoc test; * *p* < 0.05, ** *p* < 0.01, *** *p* < 0.001 and ns = not significant.

All this work was performed on hNS1 cells under the differentiation conditions (see the scheme in Figure 1A. hNS1 cells were cultured in the differentiation medium at different concentrations of Aβ40 peptide (0.5 and 1 µM). After 4.5 days of differentiation + Aβ40 treatment, the cultures were analyzed for cellular death, the proliferative rate and the phenotype specification. The presence of the Aβ40 peptide in its monomeric form was confirmed by Western blot analysis at both concentrations (0.5 and 1 µM; Figure 1B).

### 2.1. Aβ40 Peptide Effects in Cell Death in Differentiating hNS1 Cells

Aggregated forms of the Aβ peptides and their toxic effects are well-known due to their involvement in the pathology of AD [7,10,28,29]. On the other hand, the effect of the monomeric isoforms (non-aggregated or soluble forms) on NSCs is poorly understood.

As it is shown in the representative phase contrast images (Figure 1C), cell death was evident in the cells treated with the highest concentration of Aβ40 peptide (1 µM). The cells appeared damaged and with a lower density than in the other experimental groups, suggesting that this dose was toxic to hNS1 cells under these experimental conditions. To determine whether programmed cell death was involved in this effect, we analyzed activated Caspase 3 (ActCasp3) immunoreactivity, the number of fragmented nuclei and the abundance in γH2AFX for the different cellular groups.

Some enzymes such as Caspases are essential intermediaries of apoptotic/programmed cell death. Particularly, Caspase 3 is a protease important for chromatin condensation and DNA fragmentation during the apoptotic process [29].

We used an antibody that recognizes the activated form of this protease, and as shown in Figure 1D (upper panels), the immunoreactivity for activated Caspase 3 was low in the control cells but increased in parallel to the peptide concentrations, with high immunoreactivity at 1 µM.

Quantification of the percentage of ActCasp3+ cells showed that 9.1 ± 0.8% and 9.11 ± 0.7% of the total cells were positive in the control and vehicle groups, respectively. The number increased to 12.6 ± 0.8% in the 1 µM-treated group (** *p* < 0.01; *n* = 3). We did not observe any significant change in the 0.5 µM-treated group (10 ± 1%; *p* > 0.05; *n* = 3) compared to the control groups (Figure 1E).

Cells can undergo DNA condensation during programmed cell death [30,31]. In parallel cultures to those previously used, the percentage of cells with fragmented nuclei was determined after Hoechst staining. Fragmented/pyknotic nuclei appeared brighter and more disintegrated than normal nuclei (Figure 1D, bottom panels) and were quantified. The percentage of pyknotic nuclei was significantly increased after treatment with 1 µM but not with 0.5 µM (Figure 1D–F) of the Aβ40 peptide. Only 2 ± 0.3% of cells in the vehicle group presented fragmented nuclei, rising to 2.5 ± 1.1% in the 0.5 µM-treated group (*p* > 0.05; *n* = 3) and 3 ± 0.3% in the 1 µM-treated group (** *p* < 0.01; *n* = 3). These results confirmed what we observed before for the activated Caspase 3 immunoreactivity (Figure 1E).

Finally, we studied the amount of γH2AFX. H2AX is rapidly phosphorylated at serine 139 when DNA breaks occur during normal cellular processes or due to external agents [32]. As shown in Figure 1G, treatment with the Aβ40 peptide induces only a small increase in the γH2AFX abundance at the highest dose—1 µM. That suggests no clear DNA damage induction by Aβ40 in the hNS1 cells.

### 2.2. Effects of Aβ40 Peptide in Proliferation Rate of Differentiating hNS1 Cells

Many studies have observed that Aβ peptides also affect NSC proliferation [19,20,23]. In order to analyze the proliferation of hNS1 cells at different experimental doses, we used two different markers. We analyzed the expression of a nuclear protein present in all active phases of the cell division cycle but absent in resting cells (G0), so it is assumed to be expressed in proliferating cells, called the Ki67 marker [33]. Next, we detected the incorporation of BrdU in dividing cells, which is incorporated in place of thymidine into newly synthesized DNA during the S phase of the cell cycle. As shown in Figure 1H–J, 22.3 ± 1.2% of untreated control cells and 24 ± 1% of vehicle-treated cells were positive for the Ki67 marker. Treatment with the Aβ40 peptide induced a significant increase in the percentage of Ki67+ cells (Figure 1J) at the 1 µM concentration (34.2 ± 0.8%) compared to the controls (*** *p* < 0.001; *n* = 3). No significant differences were observed in the other treated group (0.5 µM, 25.8 ± 0.8%; *p* > 0.05; *n* = 3). These results were also confirmed by quantitative PCR (RT-qPCR) (Figure 1K), suggesting that 1µM has a positive effect on hNS1 cell proliferation. To detect mitotic cells, we did a short pulse (to avoid longer treatment that could affect the influence of the Aβ peptide) of BrdU for 2 h before fixation and immunocytochemistry (ICC) (Figure 1I). The results confirmed that 8 ± 1.5% of untreated cells incorporated BrdU, demonstrating that they were proliferating. After treatment with the Aβ40 peptide, a significant increase in the number of BrdU+ cells was detected at the 0.5 µM (13 ± 1.2%; ** *p* < 0.01; *n* = 3) and 1 µM (16.4 ± 1%; *** *p* < 0.001; *n* = 3) doses (Figure 1L). Taken together, these results show that treatment with the Aβ40 peptide increases the percentage of proliferating hNS1 cells, especially at the highest dose—1 µM.

### 2.3. Aβ40 Peptide Effects in Cell Fate Specification of hNS1 Cells

Several studies have pointed to the effect of Aβ peptides in neurogenesis [7,19,20]. To define the effects of Aβ peptides on neuronal specification during hNS1 differentiation, the expression of β-III-tubulin (as a marker of neurons) was analyzed in hNS1 cells incubated with freshly prepared, soluble Aβ40 peptide at the concentrations specified for 4.5 days.

**Figure 2:**
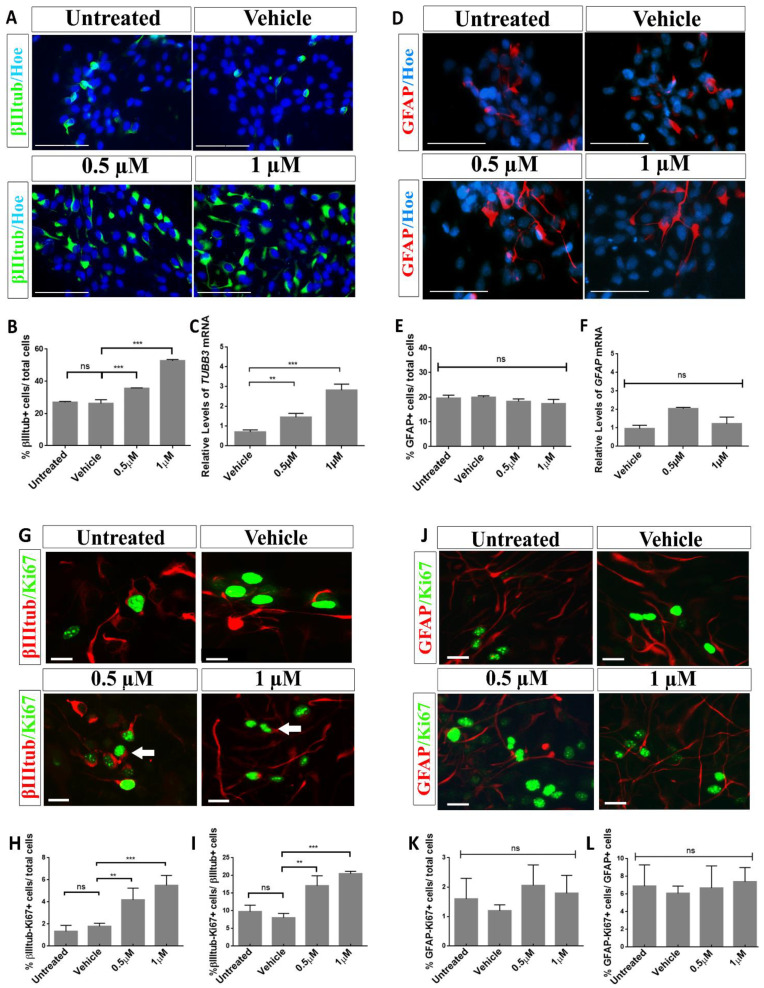
Aβ40 treatment stimulates neurogenesis in differentiated hNS1 cells. (**A**) Representative images showing β-III-tubulin immunoreactivity (βIIItub; green). (**B**) Percentage of β-III-tubulin+ cells/total cells after treatment with the Aβ40 peptide. (**C**) Relative expression levels of *TUBB3* mRNA by RT-qPCR analysis. (**D**) Images of GFAP immunoreactivity (red). (**E**) Percentage of GFAP+ cells/total cells after treatment with the Aβ40 peptide. (**F**) Relative expression levels of *GFAP* mRNA obtained by RT-qPCR analysis. Cell nuclei in (**A**,**D**) were stained with Hoechst (blue). (**G**) Representative images showing double immunoreactivity for β-III-tubulin (βIIItub; red) and Ki67 (green) (arrows). (**H**) Analysis of the percentage of β-III-tubulin-Ki67+ cells/total cells in the Aβ40-treated groups. (**I**) Percentage of β-III-tubulin-Ki67+ cells/β-III-tubulin+ cells in the Aβ40-treated groups. (**J**) Representative images showing dual immunoreactivity of GFAP (red) and Ki67 (green). (**K**) Analysis of the percentage of GFAP-Ki67+ cells/total cells after treatment with the Aβ40 peptide. (**L**) Percentage of GFAP-Ki67+ cells/GFAP+ cells after treatment with the Aβ40 peptide. Scale bars, 50 µm (**A**,**D**) 10 µm (**G**,**J**). Data are represented as the mean ± SD of at least three different experiments (*n* = 3). Statistical significance from one-way ANOVA with Tukey’s post hoc test; ** *p* < 0.01 and *** *p* < 0.001; ns = not significant.

As shown in Figure 2A,B, Aβ40 treatment significantly increased the percentage of β-III-tubulin+ cells at all doses tested (35.6 ± 0.3% in the 0.5 µM group and 51.8 ± 2% in the 1 µM group) compared to the control groups (27 ± 0.5% in the untreated cells and 26 ± 2.3% in the vehicle group) (*** *p* < 0.001; *n* = 3). These results were also validated by RT-qPCR analysis (Figure 2C), where the relative expression of *TUBB3* was significantly increased after the treatment with Aβ40 peptide compared to the control groups (** *p* < 0.01; *** *p* < 0.001; *n* = 3).

In addition, there are studies supporting the idea that Aβ peptides are involved in the stimulation of glial cell fate in AD [34], murine NSCs [19,20] and human NSCs [23,35]. Due to this, we also examined whether treatment with the Aβ40 peptide influenced the astrocyte lineage specification in differentiating hNS1 cells. After treatment, cells were analyzed for the expression of GFAP, a marker of astrocytes, and as shown in Figure 2D,E, no statistically significant changes in the number of GFAP+ cells were observed after treatment with the Aβ40 peptide. All cell groups showed approximately 20% (*p* > 0.05; *n* = 3) of the GFAP+ cells. Similar conclusions were obtained by the RT-qPCR analysis (Figure 2F), suggesting that Aβ40 does not affect gliogenesis in hNS1 cells.

Together, these results show that the Aβ40 peptide treatment of differentiating hNS1 cells increases the differentiation to a neuronal phenotype without affecting the astroglial differentiation.

### 2.4. Treatment with Aβ40 Peptide Increases Neurogenesis in hNS1 Cells under Differentiation

In this analysis, we found that Aβ40 treatment increased the proliferation and neuronal fate in differentiating hNS1 cells. To further investigate whether the effect of Aβ40 on the enhancement of lineage-specific markers was due to proliferative effects, we studied the number of cells double positive for β-III-Tubulin and Ki67 (Figure 2G). We plotted and analyzed the data with respect to the total cells (Figure 2H) and with respect to β-III-Tubulin+ cells (Figure 2I).

In the vehicle group, we observed that β-III-Tubulin+ cells were infrequently double positive for Ki67 (1.81 ± 0.6%). However, this percentage significantly increased after Aβ40 treatment, rising to 4.2 ± 0.5% (** *p* < 0.01; *n* = 3) in the 0.5 µM-treated group and to 5.8 ±1% (*** *p* < 0.001; *n* = 3) in the 1 µM-treated group, considering the group of β-III-Tubulin+/Ki67+ cells with respect to the total cells (Figure 2H). This rise in β-III-Tubulin+/Ki67+ cells supports the earlier results showing that differentiating hNS1 cells achieve an increase in proliferation and an improvement in neurogenesis after Aβ40 peptide treatment. To deepen these effects observed, we performed a further detailed study of β-III-Tubulin+/Ki67+ cells compared to the β-III-Tubulin+ cells population. Newly, we detected that groups treated with the Aβ40 peptide had a significantly increased number of β-III-Tubulin+/Ki67+ cells, increasing from 9 ± 1.5% in the control group to 16 ± 4% in the 0.5 µM-treated group (** *p* < 0.01; *n* = 3) and to 20 ± 2% (*** *p* < 0.001; *n* = 3) in the 1 µM-treated group (Figure 2I). Altogether, these results suggest an increase in the number of precursors (Ki67+) that are particularly differentiating to neuronal cells after Aβ40 peptide treatment.

To conclude whether this effect was specific to neuronal precursors, we did a similar study looking at the number of cells double positive for GFAP and Ki67 (Figure 2J). We plotted and analyzed our data against the total cells (Figure 2K) and against the GFAP+ cells (Figure 2L). We detected no statistically significant differences between the control and Aβ40 peptide-treated groups for GFAP+/Ki67+ cells. 

These data propose that treatment with the Aβ40 peptide enhances proliferation and imposes neurogenesis by increasing the pool of proliferating neuronal precursors, and this effect is neuron-specific, without affecting gliogenesis or glial progenitor levels.

### 2.5. Analysis of Possible Molecular Pathways Associated with the Effects Observed after Aβ40 Peptide Treatment

**Figure 3:**
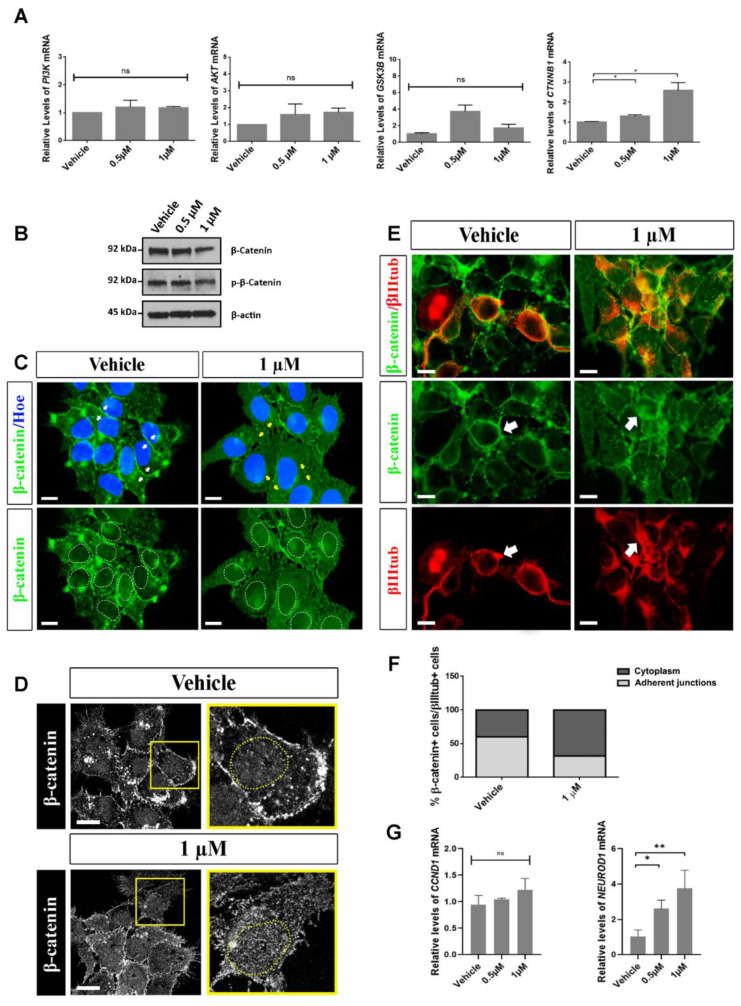
Change in β-catenin localization after treatment with the Aβ40 peptide in differentiating hNS1 cells. (**A**) Relative expression levels of *PI3K* mRNA, *AKT* mRNA, *GSK3B* mRNA and *CTNNB1* mRNA obtained by RT-qPCR analysis after treatment with the Aβ40 peptide. (**B**) WB analysis of the expression of β-catenin (92 kDa) and p-β-catenin (92 kDa) in cellular extracts after Aβ40 treatment. β-actin was used as a loading control (45 kDa). (**C**) Representative images showing β-catenin immunoreactivity (green) after Aβ40 peptide treatment for 2.5 days. Cell nuclei were counterstained by Hoechst (blue). The localization of β-catenin in adherent junctions is higher in the vehicle group (white arrows) than the 1 µM group (yellow arrows). (**D**) Representative images showing β-catenin immunoreactivity obtained by confocal microscopy. (**E**) Representative images showing double-immunoreactivity for β-catenin (green) and β-III-tubulin (βIIItub; red) after Aβ40 peptide treatment (arrows). Scale bar, 10 µm. (**F**) Percentage of β-catenin+ cells/β-III-tubulin+ cells (with immunoreactivity for β-catenin localized in cytoplasm or adherent junctions) after treatment with the Aβ40 peptide. (**G**) Relative expression levels of *CCND1* mRNA and *NEUROD1* mRNA obtained by RT-qPCR analysis after Aβ40 peptide treatment. Data are represented as the mean ± SD of at least three different experiments (*n* = 3). Statistical significance of one-way ANOVA with post hoc Tukey’s test; * *p* < 0.05 and ** *p* < 0.01; ns = not significant.

The molecular pathways affected by the Aβ peptide and, specifically, the exact role of the Aβ40 monomeric/soluble form remains poorly understood. Some authors have suggested that the Aβ peptide in its aggregated form affects many intracellular signaling pathways related to GSK3β or Ras-MAPK signaling [23,36,37]. To explore the possible molecular pathways implicated in the effects observed after Aβ40 treatment, we analyzed the expression of some genes associated with signaling pathways that could be influenced by Aβ peptides by RT-qPCR. These genes were the following: *GSK3B*, *PI3K*, *AKT* and *CTNNB1* (Figure 3A). We detected that the Aβ40 peptide treatment significantly increased the expression of *CTNNB1* mRNA (coding for β-catenin) as compared to the control groups (Figure 3A).

In addition, we performed WB assays to analyze the phosphorylation state of the β-catenin protein. In the Wnt signaling pathway, in the absence of the Wnt ligand (inactive), β-catenin is phosphorylated by a protein complex and degraded by the proteasome. On the contrary, in the presence of the Wnt ligand (active), β-catenin is not phosphorylated and is translocated to the nucleus, where it acts as a transcription factor for several target genes [38].

The results obtained do not show an increase in the phosphorylated β-catenin (p-β-catenin) levels after the treatment of hNS1 cells with Aβ40 peptide, either at a concentration of 0.5 µM or at 1 µM, where even a lower-band intensity is observed compared to the control group (vehicle) (Figure 3B). Consequently, we think that β-catenin is not preferentially degraded by the proteasome after the treatment of hNS1 cells with the Aβ40 peptide, and it could be translocating to the cell nucleus. Due to this, we performed a more extensive assay (at higher magnifications) to determine the cell localization of β-catenin after the treatment of hNS1 cells with the Aβ40 peptide (Figure 3C,D).

Unlike the control group (vehicle), where β-catenin localization is more cytoplasmic in the membrane (in adherent junctions between cells), hNS1 cells treated with Aβ40 peptide (1 µM) show a change in the cellular localization of β-catenin, being preferentially present in the cytoplasm and cell nucleus, decreasing its expression in intercellular adherent junctions (Figure 3C,D). In order to verify whether this change in the cellular localization of β-catenin could be related to the increase in neurogenesis previously observed in hNS1 cells treated with the Aβ40 peptide, we studied the cell localization of β-catenin in generated neurons (β-III-tubulin+ cells).

As can be seen in the images, the β-catenin location in the control neurons (β-catenin+/β-III-tubulin+ cells) is predominant in intercellular adherent junctions, while the location of β-catenin in the neurons generated after treatment with the Aβ40 peptide (1 µM) is mostly cytoplasmic (and even nuclear) (Figure 3E,F). Given that this change in the cell localization of β-catenin after the treatment of hNS1 cells with the Aβ40 peptide could be related to the function that it is carrying out, we analyzed the gene expression of some β-catenin target genes in order to determine if β-catenin would be playing a role as a transcriptional regulator.

The results we obtained by RT-qPCR showed an increasing trend in the *CCND1* mRNA levels (coding for cyclin D1 and involved in cell proliferation) in hNS1 cells treated with the Aβ40 peptide (1 µM), as well as a statistically significant increase in the *NEUROD1* mRNA levels (coding for NeuroD1 and involved in neuronal maturation) in cells treated with the Aβ40 peptide (0.5 µM and 1 µM) compared to the control hNS1 cells (vehicle) (Figure 3G).

To sum up, these results propose that a long exposure of high concentrations of the Aβ40 peptide could be toxic to human NSCs and activate an apoptotic pathway. Moreover, the Aβ40 peptide favors the proliferation of human NSCs undergoing differentiation and promotes a neuronal cell fate, and these effects could be mediated, at least in part, by β-catenin, which undergoes a change in cellular localization to regulate the transcription of proliferative and pro-neurogenic genes.

## 3. Discussion

AD is the most prevalent neurodegenerative disorder in developed countries for which there is still no cure. Some potentially therapeutic strategies consist of inducing the proliferation and differentiation of endogenous brain NSCs (i.e., endogenous neurogenesis) or infusion of exogenous NSCs into the brain to regenerate damaged neurons. The potential benefits of human stem cell therapy for the treatment of neurodegenerative disorders depend on the ability of implanted NSCs to survive, migrate and differentiate in the proper neural and neuronal lineages [7,10]. For this reason, a greater effectiveness is important when it comes to finding possible therapies, and it becomes necessary to deepen into the study of pathological causes in the case of AD, including the physiological and pathological effects of Aβ peptides.

Aβ peptides have been shown in some studies to have neurotrophic and/or neuroprotective functions, as they improve the survival and neurogenesis of hippocampal neurons in vitro [10,11]. That is why it is believed that monomeric Aβ peptides may possess important biological and physiological functions before aggregating into oligomers and forming fibrils [29]. Unfortunately, the cellular and molecular signals and processes that regulate these functions are poorly understood.

In this study, we demonstrated that the monomeric form of the Aβ40 peptide significantly stimulates the proliferation of human NSCs undergoing differentiation, analyzing the aspects related to alteration in cellular survival/death, cell proliferation and cell differentiation (i.e., phenotypic specification) in hNS1 cells.

Although there have been several studies describing that Aβ peptides are involved in AD pathology and its progression, there are very few data about their physiological form. Some studies have shown that the Aβ40 peptide in its monomeric form may have neuroprotective effects, so this study may help to clarify what the role of this peptide might be and the mechanisms involved in both the physiological and pathological conditions in human NSCs. Our results showed that Aβ40 provokes cell death by apoptosis at the highest dose tested (1 µM), as demonstrated by the increase in Caspase 3 activation and the percentage of pyknotic/fragmented nuclei. These results are in line with those obtained by our group for the Aβ42 peptide, where we saw that the Aβ42 peptide induces apoptotic cell death even at low doses (0.5 µM), being more toxic than the Aβ40 peptide at the same concentrations for hNS1 cells under our experimental conditions [23]. These data were also in line with previous reports observing that Aβ exhibits neuronal death at low concentrations [39,40]. It is well-known that the Aβ42 peptide is essential for the start of the Aβ deposition and generation of amyloid plaques present in the AD brain [41]. Moreover, in vitro studies have shown that the Aβ42 peptide aggregates faster than the Aβ40 peptide into oligomers and fibrils [42]. Due to this, some people consider the Aβ40 peptide an anti-amyloidogenic factor. However, although it has been mentioned above that the Aβ40 peptide seems less toxic and less prone to aggregation than the Aβ42 peptide, in our experimental conditions, we saw that these high concentrations (1 μM) of the Aβ40 peptide in its monomeric/soluble form produce an increase of apoptotic cell death.

Although the toxic effect of Aβ peptides has been described, our results and those obtained previously by our group provide evidence that the Aβ peptide promotes the proliferation of human NSC. The results showed an enhancement in the proliferative rate in hNS1 cells, as demonstrated by the increase in the percentage and expression of the Ki67 marker and by the number of BrdU-incorporating cells. Nevertheless, this increase in proliferation is less than what was previously observed for the Aβ42 peptide in these cells [23]. Previous studies [20] were in accordance with these effects, where they saw that, in mouse NSCs treated with the Aβ40 peptide (monomeric at 1.5 µM), the proliferation of these NSCs is increased. The findings presented here are in accordance with another study showing that non-aggregated Aβ40 and Aβ42 peptides increase the proliferation of NPCs isolated from fetal rat brains and that the Aβ42 peptide has a greater effect than Aβ40 peptide [19].

It should be noted that Aβ peptides are not always associated with toxicity, since, at low concentrations, the monomeric forms seem to favor neurogenesis or gliogenesis [23]. Adult neurogenesis in the CNS has an important function in learning, smell and memory [43]. In AD patients, neurogenesis is disturbed, which results in a progressive loss of brain neurons [1], partly due to the presence of extracellular aggregates of Aβ peptide. On the other hand, gliogenesis provides multiple functions to both the peripheral nervous system (PNS) and CNS, and in pathological conditions, such as AD, the gliogenesis seems to increase [44,45].

Our results showed that the Aβ40 peptide stimulates neuronal differentiation (neurogenesis) at 1 µM. In addition, we observed a growth in the percentage of proliferating precursors (Ki67+) that were also β-III-tubulin+ cells, suggesting that the proliferating precursors acquire a neuronal phenotype. Compared to the Aβ42 peptide, Aβ40 is a pro-neurogenic factor, but the concentration/dose is important. When the dose is too high, it can provoke aggregation and toxicity (mainly in the case of Aβ42). Our data and those of other authors showed that the Aβ40 peptide could be an important factor in modulating neurogenesis. In contrast, we could not detect any change in the number of GFAP+ cells or Ki67+ double-positive cells, indicating that the observed increase in the proliferation rate was specific to the increase in neuronal cells without affecting astrogliogenesis.

Regarding other studies, the functions of the Aβ peptide are highly controversial, as we can see in the results obtained from different works. Human NSCs exposed to monomeric forms of synthetic Aβ42 peptide in human NSCs showed an increase in gliogenesis without affecting the neuronal differentiation [23], while, as we mentioned before, Aβ40 promotes neurogenesis without disturbing the glial differentiation in these cells. However, these properties change when the effects of the oligomeric Aβ peptides are analyzed. Some authors have found that, in NSCs from the rat hippocampus, neurogenesis is induced by the Aβ42 peptide and not the Aβ40 peptide [46]. The reason for these discrepancies may be due to the type of peptide used in the experiments (aggregated or monomeric), the type of dose/time of exposure of the peptides or the type of cell culture used in each experiment.

Accumulating evidence suggests that β-catenin plays a key role in cadherin-based cell adhesion and the Wnt signaling pathway. Most β-catenin localizes to the cell membrane, where it associates with the cytoplasmic region of E-cadherin, a transmembrane protein involved in cell–cell contacts [38]. The Wnt/β-catenin signaling pathway is an important pathway that regulates cell proliferation and differentiation. Studies have shown that the dysregulation of one of Wnt/β-catenin signaling plays an important role in the pathogenesis of AD [47]. In fact, the loss of Wnt/β-catenin signaling makes neurons more susceptible to Aβ-induced apoptosis, and the activation of Wnt/β-catenin signaling rescues Aβ-induced neuronal death and behavioral deficits [38,47]. Our results show that the Aβ40 peptide promotes the neuronal cell fate mediated, at least in part, by β-catenin, which undergoes a change in cellular localization to regulate the transcription of proliferative and pro-neurogenic genes. To complete and conclude these results, new perspectives must be explored in order to really understand the possible mechanisms linking AB40 to the neural stem cell biology and its connection with AD, using techniques such as RNA-seq analysis.

In conclusion, we described the important effects of soluble/monomeric Aβ40 peptide on human NSCs. We found that the Aβ40 peptide increases neuronal phenotype specification during the later stages of the cell cycle. However, at high concentrations, it appears to be cytotoxic for human NSCs. With this study, we have been able to establish a better understanding of the relationships between the properties of human NSCs and Aβ peptides, showing that, at different concentrations, we are able to modulate the differentiation of human NSCs, promoting neurogenesis (Aβ40) and gliogenesis (Aβ42), while high concentrations of both peptides are cytotoxic for these cells.

It is known that the cellular and molecular effects involved in AD occur several decades before the first cognitive and clinical symptoms appear. For this reason, these results could be important in discovering new markers for earlier diagnosis and for the development of new therapeutic targets. Taken together, it can help us understand the cellular and molecular processes that happen in the brain of AD patients at the beginning of the disease. A major limitation to these studies is the lack of models, both in vitro and in vivo, that perfectly mimic an AD-affected brain [48]. However, our cell system can be a valuable tool to study the physiological context of a brain with AD and help clinical progress in stem cell-based therapies to treat this disease. Although more studies are needed, understanding the molecular mechanisms that regulates NSC proliferation and differentiation in a neurodegenerative setting may provide valuable information for potential stem cell-based therapies. However, research on potential therapeutic strategies based on the use of human NSCs for the treatment of AD has lagged far behind many other neurodegenerative disorders.

## 4. Materials and Methods

### 4.1. Ethics Statement

hNS1 cells were obtained from human tissues donated for research after written informed consent, in accordance with the European Union directives and the Declaration of Helsinki and in agreement with the ethical guidelines of the Network of European CNS Transplantation and Restoration (NECTAR) and Spanish Biomedical Research Law (July 2007). Ethics statements about the human fetal origin of the cells used here can be found in the original reports describing the cell line [24,25,26,27]. The study was approved by the Ethics Committee of the Instituto de Salud Carlos III (approval number PI93-2020, 1 December 2020).

### 4.2. Cell Cultures

Culture conditions of hNS1 cells are based on a chemically defined human stem cell (HSC) medium. For experiments, cells were seeded at 15,000 cell/cm^2^ on poly-L-lysine (10 µg/mL; Sigma, Roedermark, Germany) coated plastic dishes, and cells were grown in HSC medium supplemented with 20 ng/mL of Epidermal Growth Factor (EGF; PeproTech, London, UK) and with 20 ng/mL of Fibroblast Growth Factor 2 (FGF2; PetroTech) at 37 °C in a 5% CO_2_ incubator (Forma) [25]. HSC medium containing 0.5% heat-inactivated Fetal Bovine Serum (FBS; Gibco, Langley, OK, USA) was used for cell differentiation.

### 4.3. Preparation and Treatment with Aβ Peptide

The Aβ40 peptide in a lyophilized state (American Peptide Company, Sunnyvale, CA, USA) was dissolved in Hexafluoro-2-propanol (Sigma) into a final concentration of 1 mM. The monomeric peptide was prepared by diluting the aliquot of the dry stock of 50 μg with dimethyl sulfoxide (DMSO; Sigma) to 1 mM and then further diluting the different concentrations for analysis (0.5 and 1 µM) in differentiation medium. Culture cells were treated for the first 4.5 days of differentiation, unless otherwise specified. Untreated cells and vehicle (DMSO)-treated cells were used as controls. See the scheme in Figure 1A.

### 4.4. 5′-Bromo-2′-Deoxyuridine (BrdU) Treatment and Detection

For the detection of proliferating cells, differentiation medium containing 5 µM of 5′-bromo-2′-deoxyuridine (BrdU) (Sigma, Germany) was used for 2 h in all the experimental groups. Later, cell cultures were washed with PBS, fixed in 4% paraformaldehyde (PFA; Sigma) for 10 min, washed with PBS again and treated with hydrochloric acid 2 M (HCl; Merck, Darmstadt, Germany) for 30 min at 37 °C. BrdU is a thymidine analog and can be incorporated into newly synthesized DNA strands of mitotic cells. This incorporation of BrdU into cellular DNA can then be detected by immunocytochemistry using anti-BrdU antibody, allowing an assessment of the cell proliferative rate [49].

### 4.5. Immunocytochemistry (ICC), Image Analysis and Counting

After the specified differentiation time, cells were washed with PBS and fixed in 4% PFA for 10 min. Cell cultures were blocked for 1 h at room temperature (RT) in 0.25% Triton X-100 with 5% normal horse serum (NHS) in PBS. Cell cultures were incubated overnight at 4 °C with the primary antibodies diluted in PBS containing 0.25% Triton X-100 and 1% NHS. The following antibodies were used: mouse anti-GFAP (1:1000; BD Pharmigen), rat anti-BrdU (1:1000; Abcam (Trumpington, UK)), rabbit anti-Ki67 (1:500; Thermo Scientific (Waltham, MA, USA)), rabbit anti-β-III-Tubulin (βIIItub; 1:500; Sigma), rabbit anti-β-catenin (1:100; Cell Signaling (Danvers, MA, USA)), mouse anti-β-III-Tubulin (βIIItub; 1:2000; Biolegend (San Diego, CA, USA)) and rabbit anti-activated Caspase 3 (Casp3; 1:500; Cell Signaling). Cultures were incubated for 1 h at RT with one of the corresponding secondary antibodies: Alexa Fluor 555 donkey anti-mouse IgG, Alexa Fluor 555 goat anti-rat IgG and Alexa Fluor 488 donkey anti-rabbit IgG (1:500; Life Technologies (Carlsbad, CA, USA)). Cell nuclei were counterstained with Hoechst 33258 (Hoe; Invitrogen (Waltham, MA, USA)) diluted in PBS (1:1000) for 5 min at RT.

Analysis and photography of fluorescent cultures were done using a confocal microscope (Leica SP5) and a fluorescence microscope (Leica DM IL LED) coupled to a camera (Leica DFC 345 FX). At least 8 fields per well were randomly acquired at 40× magnification to quantify the number of positive cells for the different markers compared to the total number of cells (Hoechst). Each marker was studied in at least 3 different wells of the same experiment, and each experiment was repeated 3 independent times (*n* = 3). Cell counting was done using the program ImageJ (National Institute of Health) and Adobe Photoshop CS6.

### 4.6. RNA Isolation, cDNA Synthesis and Quantitative PCR (RT-qPCR)

Total RNA was isolated with the RNeasy Mini extraction kit (Qiagen, Hilden, Germany), according to the manufacturer’s instructions. One microgram of total RNA was reverse-transcribed at 50 °C for 60 min in a 20-µL reaction mixture using SuperScriptIII-RT (Life Technologies, (California, USA). Relative amounts of cDNA were quantified by quantitative real-time PCR (qRT-PCR) using the FAST SYBR-green system (Applied Biosystems, Waltham, MA, USA), according to the manufacturer’s protocols. Each 15 µL reaction volume included 10 ng total cDNA and 0.3 µM of each primer. qRT-PCRs were performed using primers for the following human target genes: *GFAP* (forward: 5′-GTTCTTGAGGAAGATCCACGA-3′; reverse: 5′-CTTGGCCACGTCAAGCTC-3′), *TUBB3* (forward: 5′-GCAACTACGTGGGCGACT-3′; reverse: 5′-ATGGCTCG AGGCACGTACT-3′), *KI67* (forward: 5′-TGACCCTGATGAGAAAGCTCAA-3′; reverse: 5′-CCCTGAGCAACACTGTCTTTT-3′), *GSK3B* (forward: 5′GAAAGTATTGCAGGACAAGAGATTT-3′; reverse: 5′-CGGACTATGTTACAGTGATCTAGCTT-3′), *PI3K* (forward: 5′-GGCTCAAAGACAAGAACAAAGG -3′; reverse: 5′-TCCAGCACATGAACGTGTAAA-3′), *AKT* (forward: 5′-GGCTATTGTGAAGGAGGGTTG-3′; reverse: 5′-TCCTTGTAGCCAATGAAGGTG-3′), *CTNNB 1* (forward: 5′-CAAGTCCAAGATCAGCAGTCTC-3′; reverse: 5′-GCTTTCAGTTGAGCTGACCA-3′), *NEUROD1* (forward: 5′-CTGCTCAGGACCTACTAACAACAA-3′; reverse: 5′-GTCCAGCTTGGAGGACCTT-3′), *CCND1* (forward: 5′-GCTGTGCATCTACACCGACA-3′; reverse: 5′-TTGAGCTTGTTCACCAGGAG-3′) and housekeeping gene *TBP* (forward: 5′-GAGCTGTGATGTGAAGTTTCC-3′; reverse: 5′-TCTGGGTTTGATCATTCTGTAG-3′). The Applied Biosystems 7500 Real-Time PCR System was used to determine the amount of target mRNA in each sample, estimated by the 2^−ΔΔCt^ relative quantification method [50]. Gene expression levels were normalized against TBP levels in each sample, and the fold change was calculated by setting the expression levels of each gene in the vehicle (DMSO) control as 1.

### 4.7. Western Blot (WB)

Differentiation medium with Aβ40 peptide treatment for each condition was collected and analyzed by WB in order to determine the presence of the Aβ40 peptide in its monomeric form. For the detection of cell death, β-catenin and p-β-catenin, 50 µg of cellular extracts of differentiated cultures after Aβ peptide treatment were analyzed. Samples were boiled for 5 min, loaded on a 12% sodium dodecyl sulphate (SDS)-polyacrylamide gel, electrophoresed and transferred to nitrocellulose membranes (GE Healthcare). Membranes were blocked in PBS containing 5% nonfat dairy milk with 0.05% Tween20 (Sigma) for 1 h at RT. Blots were incubated overnight at 4 °C with primary antibodies against mouse β-actin (1:1000; Sigma), mouse anti-Aβ 4G8 (1:1000; Covance) mouse anti-phospho-Histone H2A.X (γH2AFX; 1:1000; Millipore (Burlington, MA, USA)), rabbit anti-β-catenin (1:1000; Cell Signaling) or rabbit anti-phospho-β-catenin (1:1000; Cell Signaling). The blots were developed using peroxidase-conjugated goat anti-rabbit (GARPO; 1:3000; Vector Laboratories), peroxidase-conjugated horse anti-mouse (HAMPO; 1:3000, Vector Laboratories) or goat anti-rabbit (GARPO; 1:3000; Vector Laboratories) for 1 h at RT and visualized using the ECL system (Millipore).

### 4.8. Quantification of Fragmented/Pyknotic Nuclei

Those cells that exhibit the morphological hallmarks of apoptosis, such as nuclear fragmentation, are defined as apoptotic cells. At least 8 fields per well were randomly acquired at 40× magnification to quantify the number of positive cells for the fragmented nuclei compared to the total cells (Hoechst). Cell counting was done using the program ImageJ (National Institute of Health) and Adobe Photoshop CS6.

### 4.9. Statistical Analysis

Statistical analyses were run using GraphPad Prism 6.0. Mean values were compared using one-way ANOVA (Analysis Of Variance) with post hoc Tukey’s test. *p*-values < 0.05 were statistically significant (* *p* < 0.05, ** *p* < 0.01 and *** *p* < 0.001; ns = not significant). Results are shown as the mean ± SD of data from three independent experiments (*n* = 3), with at least 3 samples per experimental group.
